# Applications of Systems Science to Understand and Manage Multiple Influences within Children’s Environmental Health in Least Developed Countries: A Causal Loop Diagram Approach

**DOI:** 10.3390/ijerph18063010

**Published:** 2021-03-15

**Authors:** Claire F. Brereton, Paul Jagals

**Affiliations:** Children’s Health and Environment Programme, Child Health Research Centre, University of Queensland, Brisbane, QLD 4101, Australia; claire.brereton@uq.edu.au

**Keywords:** children’s environmental health (CEH), least developed countries (LDC), systems science, systems thinking, causal loop diagram (CLD)

## Abstract

Least developed countries (LDCs) are home to over a billion people throughout Africa, Asia-Pacific, and the Caribbean. The people who live in LDCs represent just 13% of the global population but 40% of its growth rate. Characterised by low incomes and low education levels, high proportions of the population practising subsistence living, inadequate infrastructure, and lack of economic diversity and resilience, LDCs face serious health, environmental, social, and economic challenges. Many communities in LDCs have very limited access to adequate sanitation, safe water, and clean cooking fuel. LDCs are environmentally vulnerable; facing depletion of natural resources, the effects of unsustainable urbanization, and the impacts of climate change, leaving them unable to safeguard their children’s lifetime health and wellbeing. This paper reviews and describes the complexity of the causal relationships between children’s health and its environmental, social, and economic influences in LDCs using a causal loop diagram (CLD). The results identify some critical feedbacks between poverty, family size, population growth, children’s and adults’ health, inadequate water, sanitation and hygiene (WASH), air pollution, and education levels in LDCs and suggest leverage points for potential interventions. A CLD can also be a starting point for quantitative systems science approaches in the field, which can predict and compare the effects of interventions.

## 1. Introduction

Children can be considered as least developed countries’ most valuable resources, but in least developed countries (LDCs), their health is threatened by ecological degradation, pervasive inequalities, climate change, migration, and urbanisation [1,2,3,4]. There are currently 46 LDCs, and these are home to over a billion of the world’s people [5]. LDCs are diverse in geography, topography, and climates, and include mountainous countries such as Nepal, tropical Pacific Island countries, and arid landlocked countries such as Mali. However, they share common characteristics of low per capita income, an economy dominated by subsistence activities, limited manufacturing, and an undiversified production structure, low education levels, high fertility rates, and inadequate infrastructure [6]. In six LDCs, more than 70% of the population live below the international poverty line [7].

LDCs account for 13% of the world’s population, but with birth rates averaging 4.2 children per woman, they will account for 45% of the global population growth by 2050. Whilst two-thirds of people in LDCs still have rural subsistence lifestyles, urbanisation rates are higher than the global average [8], with urban migration driven by rural poverty and climate change [9]. Whilst global under-five mortality rates have decreased by 59% since 1990 [10], morbidity related to early-life environmental exposures is increasing [11]. Direct and indirect effects of detrimental environmental exposures in childhood often persist through adulthood [12,13,14,15,16], affecting people’s lifetime health and wellbeing and their ability to contribute economically to their community and society. The future economic potential of LDCs is thus directly linked to the health of their children.

Children’s environmental health (CEH) is the study of how environmental exposures in early life influence health and development in childhood and the entire lifespan [17]. In two landmark reports, Preventing Disease through Healthy Environments and Healthy Environments for Healthy People, the environment is defined as all the physical, chemical, and biological factors external to a person and all the related behaviours [18,19]. Such definitions, whilst they recognise that social determinants are closely linked to vulnerability to environment, may have over time contributed to a general perception of environmental health (EH) as a discipline that focuses on modifiable physical, chemical, and biological environmental determinants of health within constructs such as water, sanitation and hygiene (WASH), air pollution, chemical use, etc. In this paper, we use the terms “environment” or “environmental” to refer to the physical, chemical, and biological environment and refer to it as a domain whilst also considering social and economic domains and their influences on children’s health outcomes in LDCs. A domain is used in the non-specialist sense to mean a sphere of activity or knowledge. For the purposes of this paper, EH is therefore defined by the social, economic, and technological influences that link environmental conditions to human health.

The science of systems thinking studies how component parts in a system connect, react, and interact and helps us to see the forest as well as the trees. It increases our capability to recognise that cause and effect are non-linear, that the outcome of an event can influence the cause, and that perceived problems can often be symptoms of other problems [20,21,22]. In systems thinking terminology, children’s health in LDCs and the environmental, social, and economic factors that influence it are the CEH system, the product of the interactions between a set of parts that influence and feed back into one another to function as a whole. Whilst systems science has been used extensively in fields such as environmental science and business, the application of its techniques has been limited in EH [23,24,25]. Systems thinking can be contrasted with linear thinking, which assumes that a cause leads to an effect with no feedbacks and that factors are independent. A major shortcoming of linear thinking is that interventions can have unintended consequences; for instance, the use of agri-supporting products like fertilisers and biocides leads to resistant pests and weeds as well as excessive nutrient enrichment of receiving environments. This leads to a drastic decline of natural ecosystems, accumulations of toxins in food chains, and pathogen resistance. This can be attributed to policies and practices not considering the feedback loops in our system, which may change the outcomes of what we try to achieve.

The CEH system in LDCs is complex and multifaceted. One common characteristic of complex problems is that the root problem that is causing the symptoms is not always apparent at first inspection, nor is the solution obvious once the problem has been defined [26]. A systems thinking approach towards understanding the feedback loops may provide new insights and help to determine root causes.

A causal loop diagram (CLD) is a qualitative systems science tool that shows the relationships between a set of variables (factors liable to change) operating in a system. It is a powerful tool for identifying the non-linear feedback loops that operate in the system to amplify or balance outcomes. It can help stakeholders to converge on a shared mental model of a system, a set of beliefs, values, and assumptions that underly why things work as they do [27]. This shared understanding about how something works and what is important can be used to enhance policy setting and decision-making. A CLD can also be the foundation for quantitative modelling techniques such as dynamic and agent-based modelling [28].

Many studies have reported on children’s health and the environment, but it appears that none have used a CLD (also known as an influence diagram) [28] to represent the big picture, the underlying feedback mechanisms and potential key leverage points. The objectives of this paper are firstly to represent the major feedback loops that link children’s health with the environmental, social, and economic domains in LDCs and secondly to seek insights into potential leverage points and interventions.

In the Results section, the CEH system is represented by a CLD that contains four interlinked sections; children’s health outcomes and the variables that influence them grouped into environmental, social, and economic domains. These domains align with the three pillars of sustainable development on which the UN Sustainable Development Goals (SDGs) are based; environmental, social, and economic [29,30].

## 2. Methodology

We used the Institute for Health Metrics and Evaluation (IHME) Global Burden of Disease results tool for 2019 [31] to identify the most significant estimated causes of child mortality and morbidity for countries in the World Bank’s least developed countries category and summarised the findings. We conducted a narrative review [32] of papers retrieved following a systematic search of current and past literature. The results were summarised, in a table for children’s health outcomes and three tables for influencing factors from environmental, social, and economic domains. These tables were used as the basis for constructing the CLD. The most important loops, based on their relative contribution to child morbidity and mortality, were then further investigated using the CLD and potential leverage points for solutions identified in Results Section 3.5.

### 2.1. Literature Review

Information on CEH in LDCs is found in reports from studies focusing specifically on LDCs, also in low- and middle-income countries (LMIC) and global health studies. Reports and scientific papers published from January 2000 through December 2020 were searched, screened, and reviewed according to their relevance, based on the primary and secondary key terms.

Key search terms were developed to ensure that potentially relevant studies with content relating to children’s health in LDCs were identified. First, searches were run using a composite primary search term “least developed country” OR “least developed countries” OR “LDC” OR “LDCs” OR “low-income countries” OR “low- and middle-income countries” OR “LMIC” with secondary search terms of “children’s health”, “environmental health”, “children’s environmental health”, and “CEH”. The composite primary search term was next used with secondary search terms taken from the causes of child mortality and morbidity identified in Table 1, e.g., “respiratory”. A further search was run using the term “global children’s health”. Databases searched were PubMed, Google Scholar, and the World Health Organization (WHO), United Nations International Children’s Emergency Fund (UNICEF), United Nations Environment Programme (UNEP), and World Bank publications databases. The same series of search terms were then used in the Google search engine to identify additional grey literature. For all sources, the first 100 results were checked, and a relevancy assessment approach [32] was used. References from identified publications were also searched.

### 2.2. Causal Loop Diagram Principles

A CLD consists of variables and cause-effect links (also known as influencing links) that connect to form causal loops, also known as feedback loops. Causal loops are either reinforcing (vicious or virtuous circles) or balancing, where self-correction occurs within the system. Every causal loop tells a story that links cause and effect through feedback, e.g.,

- reinforcing—a dengue fever epidemic where the number of infected mosquitos drives up the number of infected humans, which in turn increases the number of infected mosquitos;
- balancing—where sweating is initiated in response to heat to regulate human body temperature.

The variables that represent the causal influences in the CLD are linked by directional arrows, which represent causal associations. Associations are either:

- reinforcing—denoted by a +, in which an increase in a variable causes an increase in the variable it influences and vice versa, or e.g., internal air pollution increases respiratory disease;
- opposing—denoted by a −, when an increase in a variable causes a decrease in the variable it influences and vice versa, e.g., a clean water supply decreases WASH-related disease [28].

An even number of negative polarities in a loop denotes a reinforcing loop; an odd number, a balancing loop. Hash marks on the connector arrows denote delays between cause and effect. Variables in a CLD are either endogenous, both influencing and influenced by other variables within the CLD, or exogenous, influencing but not being influenced [22]. A further explanation of the notation of causal loop diagrams with examples is given in Supplementary Material S1.

### 2.3. Table and Causal Loop Diagram Creation

Children’s health outcomes and their influencers as identified by the literature review were all designated as variables for the CLD and grouped into four sections: children’s health outcomes, environmental, social, and economic domains. A table was created for each section, and each variable was mapped to:

- variables that it directly influences;
- variables that it is directly influenced by.

The mapping process identified two exogenous variables, remoteness and climate change, which are discussed in Section 3.3.2.

These tables were used as the basis for constructing the CLD, which was created using Stella Architect software (iseesystems.com; Version 2.0.3). The CLD is a visual representation of the mapping shown in the tables, with many loops, both reinforcing and balancing, identified. The CLD and tables were then reviewed and refined using an iterative process. The most important loops in the CLD, based on their relative contribution to child morbidity and mortality as identified in Table 1, were then further investigated and potential leverage points for solutions were identified.

## 3. Results

### 3.1. Child Mortality and Morbidity in LDCs

Table 1 shows the disease groups that contribute the most to child mortality and lifetime morbidity as measured by deaths and years lived with disability (YLD) for diseases originating in childhood. The health data are categorised by Level 2 ICD codes [31]. Childhood is defined as ages 0–14 (inclusive) in line with the definition used in the SDGs [33].

The most common cause of childhood death in LDCs, in common with global rankings, is neonatal disorders, reflecting the high-risk 28-day post-natal period. Enteric disease followed by respiratory disease and neglected tropical diseases (NTDs) are the next highest ranked. The largest contributor to lifetime morbidity is nutrition-related disease, followed by skin and subcutaneous disease, which is prevalent in LDCs. The rankings are similar for under-five mortality and morbidity, with the largest discrepancy between under-fives and under-fifteens in lifetime morbidity caused by mental disorders, which are likely to be undiagnosed in under-fives.

### 3.2. Exclusions from CLD Scope

Table 1 shows that disease groups for HIV/AIDS, sense organ diseases, and neoplasms contributed 3% or less to only one of mortality or morbidity. They were excluded from the scope of the CLD because of their relatively small contribution to child health outcomes relative to other disease groups. Disease groups for other NCDs and mental disorders were excluded from further analysis because the literature review did not yield sufficient evidence for a relationship between the disease and the environmental, social, and economic domains to warrant their inclusion in the CLD.

### 3.3. Influencing Linkages

**Figure 1:**
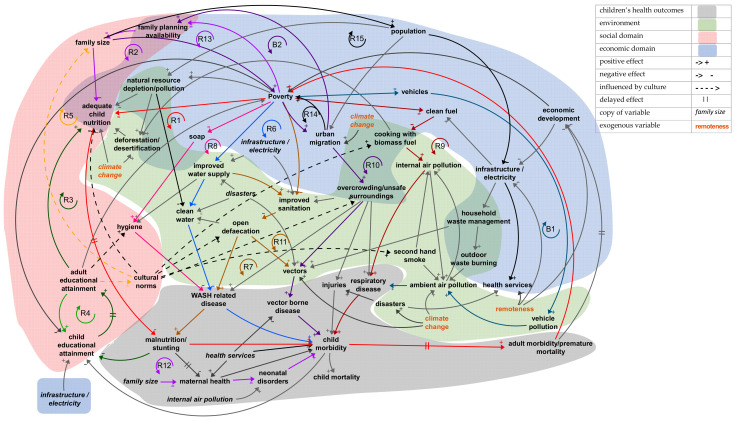
Causal loop diagram for children’s environmental health system.

Table 2, Table 3, Table 4 and Table 5 show the linkages of variables with their influencers and influences. The tables show the cause and health effect pathways (e.g., internal air pollution leads to childhood respiratory disease) and also show links between variables such as economic development, poverty, infrastructure, clean water access, morbidity, and poverty, which are then represented visually in the CLD shown in Figure 1. Table notation and logic are as follows:

- Variables are described in the shortest form possible, e.g., vehicles means number of vehicles, family size means number of people in the biological family, and clean water means the availability of a clean water supply;
- Polarities of links are shown, e.g., open defaecation increases WASH-related disease;
- Each endogenous variable influences other variables in the tables and is in turn influenced by other variables. As an example, WASH-related disease is the variable in the first row of Table 2. It appears as an influencer (reinforcing or positive) of malnutrition/stunting in the second row of Table 2 and is shown as being influenced by open defaecation in Table 3;
- The relationship between the variable and the links appearing in the “influenced by” column is summarised in the table text with supporting references. Relationships for items in the influences column are summarised when they appear in the variable column, usually in another table;
- Only direct influencers are shown, e.g., the influence of improved sanitation on WASH-related disease is not shown in Table 2, as improved sanitation is categorised as an influencer of open defaecation and can be found in Table 3.

#### 3.3.1. Health Outcomes

Children’s health outcomes in LDCs are grouped in Table 2 according to the main environmental influences; thus, the WASH-related disease category includes enteric disease, skin disease, and parasitic diseases. Influences that generally improve child health outcomes, e.g., access to health services, are linked to a consolidated child morbidity/mortality outcome. Adult morbidity and premature mortality are also influenced by child morbidity.

#### 3.3.2. Environmental Domain

The environment in LDCs is heterogeneous, with wide variation in geography, climate, and population density. LDCs contain both urban population concentrations and remote rural settlements. Endogenous variables in this domain that influence children’s health in LDCs are shown in Table 3. Two exogenous variables were identified in the environment: climate change and remoteness. They can be seen in the “influenced by” column of Table 3. In practical terms, exogenous variables cannot be influenced by other variables in the model. This means that in the context of this model, our focus for interventions should lie elsewhere.

- LDCs contribute only 0.5% of the annual carbon dioxide emissions that are driving climate change, producing 0.17 million kt of a global annual total of 34 m kt [57]. Climate change impacts are specific to individual countries and regions, but all LDCs are vulnerable to the effects of climate change, manifested in rising temperatures, changing landscapes, and increased magnitude and frequency of natural disasters [52]. Climate change has thus been treated as an exogenous variable, influencing but not being influenced by the other variables in the CLD [22].
- Remoteness is part of the economic vulnerability index for LDCs, calculated as an indicator of distance from world markets [59], and is a structural obstacle to the development of adequate infrastructure. Whilst an LDC can improve its infrastructure and services, it cannot change its geographical remoteness, which is thus an exogenous variable.

**Table 3:** Variables in environmental domain with influencing links.

Variable	Influenced by	+/−	Influences	+/−	Source
**Improved water supply**—delivered through water services infrastructure. Poverty prevents community-based improvement of water supplies when not provided by the state. Disasters affect water supply by damaging infrastructure and by flooding, which leads to contamination of both natural and improved water supplies.	Infrastructure Poverty Disasters	+ − −	Clean water Hygiene	+ +	[3,18,52,54,60]
**Clean water**—In urban/peri-urban areas, polluted watercourses are the only water source for many. Clean water is depleted through open defaecation and pollution of natural resources through activities such as logging or mining. In rural areas, clean water may be available from natural sources such as springs but may be too far from dwellings to be easily accessible.	Improved water supply Open defaecation Natural resource depletion/pollution Disasters	+ − − −	WASH-related disease	−	[3,18,52,54,60]
**Improved sanitation**—is generally dependent on an improved water supply and adequate investment in infrastructure to build and maintain it. Poverty prevents community-based sanitation improvements. Overcrowding in urban/peri-urban settings acts against sufficient improved sanitation.	Improved water supply Infrastructure Poverty Overcrowding	+ + − −	Open defaecation	−	[3,18,41,54,61,62]
**Open defaecation**—is the norm for up to 70% of people in some LDCs, both in rural and peri-urban settings. In addition to providing access to adequate sanitation, behaviour change, influenced by education, is required to effect optimal use of sanitation and to overcome cultural norms and/or taboos.	Improved sanitation Cultural norms	− +	Clean water Vectors WASH-related disease	− + +	[3,18,41,54,61,62]
**Vectors**—open waste dumps, pooled surface waters, and open faeces in LDCs attract insects and rodents, especially in overcrowded settings. Climate change-related temperature rise increases vector breeding sites.	Open defaecation Household waste management Climate change *	+ − +	Vector-borne disease	+	[51,63,64]
**Overcrowding**—mostly in urban/peri-urban areas. Unsafe surroundings are often associated with a poor quality built environment and slum dwellings with insecure structures. Culture in some LDCs requires extended family to house migrating relatives, which increases overcrowding.	Poverty Urban migration Cultural norms	+ + +	Injury Respiratory disease Skin diseases	+ + +	[18,39,49]
**Internal air pollution (IAP)**—caused by biomass cooking and lighting fuel, including wood and kerosene. Second-hand smoking and AAP that enters the home also contribute to IAP.	Biomass cooking Second-hand smoke AAP	+ + +	Respiratory disease Ambient air pollution	+ +	[3,18,65]
**Second-hand smoke**—smoking is an established part of the culture in many LDCs.	Cultural norms	+	IAP	+	[3,18,65]
**Ambient air pollution (AAP) and vehicle pollution**—in urban environments with concentrated populations, open burning of waste as well as vehicle traffic contribute to AAP. IAP becomes AAP. LDCs rarely have effective pollution controls and are likely to import ageing vehicles that no longer comply with richer countries’ stricter emission standards. Poorly surfaced roads create dust pollution. Rising temperatures due to climate change increase AAP.	IAP Outdoor waste burning Vehicle pollution Climate change *	+ + + +	Respiratory disease	+	[18,34,65,66]
**Household waste management**—is often inadequate in LDCs, particularly in urban areas, due to lack of infrastructure and funding, leading to household and other waste burning.	Infrastructure	+	Vectors Outdoor waste burning	− −	[67]
**Natural resource depletion and pollution**—is caused by increased population pressure, overexploiting resources beyond their sustainable limits (e.g., harvesting wood for fuel), and poor waste management. Pollution compromises natural water supplies.	Population Cooking with biomass fuel	+ −	Deforestation/desertification Adequate child nutrition Clean water	+ − −	[68,69]
**Deforestation/desertification**—is caused by natural resource depletion and accelerated by climate change.	Climate change *	+			[68,69]
**Natural disasters**—are increasing in frequency and magnitude. Many LDCs are in disaster-prone areas and do not have resources to mitigate the effects of natural disasters due to both inadequate infrastructure and inadequate disaster response resources	Climate change *	+	Injury Vectors Clean water Urban migration	+ + − +	[70,71]

* Exogenous variable.

#### 3.3.3. Social Domain

The social domain encompasses the social relationships and cultural constructs within which people function and interact. Components of the social domain include cultural and religious beliefs and practices, family structures, social and power relations, and inequalities. Social domain components function at multiple scales: households, extended kin networks, communities, and cities. Social domains are dynamic and change over time [72]. Variables in the social domain that influence children’s health in LDCs are family size, education levels, and culture, as shown in Table 4.

The term “cultural norms” has been used in the tables to denote the set of beliefs and practices that influence many aspects of life in LDCs. Some examples are food preparation and cooking practices, acceptability of smoking, and views on the optimum number of children for women. Cultural norms may support or be detrimental to children’s health. They will change over time, driven by influences such as education. In Table 3, cultural norms are represented as a force that resists and slows down positive change. In general, higher levels of adult education reduce the strength of detrimental habits and taboos and improve cultivating of health-supporting behaviours, for instance, by optimising sanitation and hygiene practices if water infrastructure and services are available. Without education, low adoption or declines in usage occur as communities revert to their traditions of open defaecation [23]. Cultural norms can also be seen influencing variables in both the environmental and economic domains shown in Table 3 and Table 5.

**Table 4:** Variables in social domain with influencing links.

Variable	Influenced by	+/−	Influences	+/−	Source
**Child education**—is reduced by poor health and malnutrition and exposure to biomass fuel, which affect cognitive ability. It is impaired by poverty and by the expectations and priorities of the child’s carers, who may prioritise work or care of younger children over a child’s education, particularly for females. In rural areas, a lack of electricity and low access to schools reduce study opportunities.	Poverty Adult education Child morbidity/malnutrition Electricity	− + − +	Adult educational attainment	+	[45,73,74,75]
**Adult educational attainment**—adult education levels are improved as better educated children grow into adults.	Child education	+	Malnutrition/stunting Family size Hygiene	− − +	[59,76]
**Cultural norms**—cultural norms and behaviours that affect many influencers of children’s environmental health (CEH) are influenced by education levels. Examples are the inverse relationship between adult female education level and family size and cooking with biomass fuel, an established tradition in most LDCs. Similarly, open defaecation in rural settlements is associated with privacy and comfort in many LDCs, and a move to improved sanitation can only be made with both infrastructure in place and a change of cultural norms.	Adult educational attainment	−	Family size Adequate child nutrition Open defaecation Cooking with biomass fuel Second-hand smoke Overcrowding/unsafe surroundings	− − + + + +	[76,77]
**Family planning availability**—LDCs have both low contraceptive use (39%) and a high unmet need for family planning (22%). Poverty is associated with a lack of access to modern family planning services, whereas living in urban areas is associated with better access.	Poverty Urban migration	+ −	Family size	−	[78]
**Family size**—cultural norms and expectations, reinforced by the requirement for children to support their parents, keep birth rates high, particularly in rural areas where the cost of raising a child is low and access to modern family planning services is limited or non-existent.	Family planning availability Cultural norms	− +	Population Adequate child nutrition	+ −	[78,79]
**Hygiene**—handwashing and hygienic food preparation require an improved water supply close to the home. The definition of a basic water supply is up to 30 min round trip, which is not conducive to hygienic habits. Adult education, both general and WASH specific, is a prerequisite for the establishment of hygiene in families/communities.	Improved water supply Soap Adult educational attainment	+ + +	WASH-related disease	−	[60]

#### 3.3.4. Economic Domain

Children’s health is directly affected by their economic status, with clear evidence of influencing links between economic status and EH assets such as clean water, sanitation, clean fuel, and electricity [80,81]. Table 5 shows the variables in the economic domain that influence children’s health in LDCs, including the availability of health services and urban migration driven by rural poverty.

**Table 5:** Variables in economic domain with influencing links.

Variable	Influenced by	+/−	Influences	+/−	Source
**Poverty**—low economic development leads to limited individual economic opportunity in LDCs. This is reinforced by low levels of education and large family sizes, which drain resources and increase the family income needed to live above the poverty line. Illness and premature death, with their origins in childhood morbidity, deprive families of their breadwinners or require discretionary but often non-existent health expenditure. Rural poverty drives urban migration, which also reinforces poverty due to higher food and living costs and inadequate infrastructure, particularly in informal settlements.	Economic development Adult morbidity/premature mortality Adult educational attainment Urban migration Family size	− + − + −	Urban migration/overcrowding Adequate child nutrition Lack of access to clean fuel Improved sanitation/soap	+ − + −	[54,82,83]
**Economic development**—influenced by the remoteness that characterises LDCs and by adult health outcomes. There are many other influences outside the scope of this study that could form an economics-focused causal loop diagram (CLD).	Remoteness * Adult morbidity/premature mortality	− −	Poverty Infrastructure/electricity	− +	[56,58,59]
**Adequate child nutrition**—has different influencers in urban and rural settings. Living below the poverty line does not in itself deny children access to sufficient nutrition as long as natural resources can support food production or hunting/fishing. Large family sizes make consistent availability of food more difficult, particularly in urban areas where a subsistence lifestyle cannot be practised. In some LDCs, cultural practices mean that food priority is not given to children.	Poverty Family size Natural resource depletion/pollution Adult education Cultural norms	− − − + −	Malnutrition/stunting	−	[44,45,77,83,84]
**Soap**—food takes priority over soap when there are limited financial resources.	Poverty	−	Hygiene	+	[18]
**Urban migration**—LDCs are less urbanised than their more developed counterparts, with an average of 70% of their populations living in rural settings, but their urbanisation rates are higher than global averages as rural poverty and climate change drives families to cities, often to live in urban or peri-urban informal settlements with poor infrastructure.	Poverty Climate change *	+ +	Overcrowding Poverty Family planning availability Family size	+ + + −	[9,79,85]
**Infrastructure/electricity**—which includes transport, WASH, and health facilities, is caused by low funding and compounded by the remoteness of many LDCs. Infrastructure is easily damaged by disasters, which are increasing as the impacts of climate change worsen. Population growth does not affect the absolute level of infrastructure and electricity investment but does influence its per capita availability. Small-scale solar electricity is still economically out of reach of many people in LDCs.	Economic development Remoteness * Population growth	+ − −	Health services/clean fuel/improved water Household waste management	+ +	[70,86]
**Health services**—many rural children in LDCs can only reach health facilities on a planned trip, if at all, leaving them vulnerable in emergency situations. Rural health facilities are short of qualified personnel, essential supplies, and medicines and may have no electricity, compromising the cold chain. Urban resources are stretched by growing populations. Items such as mosquito nets are in short supply.	Infrastructure Remoteness *	+ −	Child morbidity/mortality	−	[7,81]
**Clean fuel**—rural poor cannot afford clean alternatives to biomass fuels such as wood, which they can collect for free but at great personal costs in time and distance travelled. Alternatives are unavailable if there is no electricity or distribution infrastructure.	Poverty Infrastructure/electricity	− +	Cooking with biomass fuel	−	[6,7]
**Cooking with biomass fuel**—cultural as well as financial barriers must also be overcome to change behaviours to move towards clean cooking methods.	Clean fuel Cultural norms	− +	IAP	+	[18,87]
**Vehicles**—as poverty reduces, vehicle numbers, particularly in urban areas, grow.	Poverty	−	Vehicle emissions	+	[4,7,88]

* Exogenous variable.

### 3.4. Overall Causal Loop Diagram

The CLD shown in Figure 1 represents the non-linear causal relationships in the children’s health system in LDCs based on the relationships identified in the literature review. It has been structured into four sections: health (grey), environment (green), social domain (pink), and economic domain (blue). Where variables can be categorised in more than one section, e.g., overcrowding, which could be viewed as both an environment and an economic variable, the colours overlap. Many causal loops can be identified, reflecting the complexity of the system. All of the loops that include children’s health outcomes include variables in at least two other sections, showing their interconnectedness. The majority of the many loops in this diagram show reinforcing cycles, causing accelerated growth or decline. Interventions discussed later can be used to change the direction of these causal loops.

The balancing and reinforcing loops considered to be most important are shown on the CLD, but its complexity makes it hard to trace the connections, so they are split out and discussed in Section 3.4. Connections that are discussed are shown in colour or in black, e.g., blue from clean water to WASH-related disease. All others are shown in dark grey.

The term cultural norms as described in Section 3.3.3 describes a very broad range of human behaviours and customs. The table entries and the CLD show links from cultural norms to variables not only in the social domain but also to variables in the environmental and the economic domain. The CLD represents the links from cultural norms to variables in the environmental, social, and economic domains with dotted connectors to recognise that they are generalised and may not apply in all LDCs.

### 3.5. Analysis of Causal Loops

Areas of the CLD for more detailed analysis were chosen by referencing Table 1 and selecting the loops that include the disease groups, which cause the largest percentages of child mortality and lifelong morbidity. A loop focusing on the effects of population growth on children’s health was also added after linkages were noted in Section 3.5.1 and Section 3.5.5. Reinforcing and balancing feedback loops are highlighted and discussed. The loops can all be traced in Figure 1, but some positions have been rearranged for ease of reading.

#### 3.5.1. Nutritional Deficiency Loops

**Figure 2:**
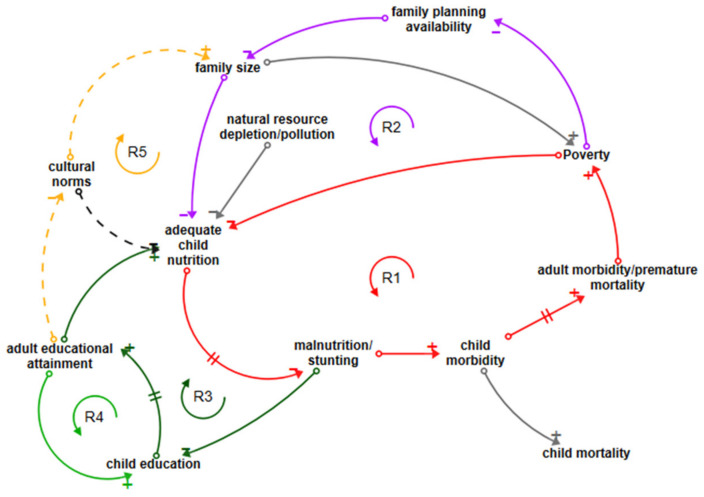
Loops influencing nutritional disease.

The most significant cause of lifetime morbidity from diseases contracted in childhood is nutritional deficiencies, primarily including protein/energy malnutrition, with 26.6% of all morbidity caused by this disease group [31]. Loop R1, shown in red in Figure 2, shows the reinforcing cycle of poverty, which reduces a family’s ability to provide adequate child nutrition. A reduction in adequate nutrition leads, with a cumulative and delayed effect, to malnutrition and/or stunting, which in its turn reinforces child morbidity [2,18,43,44]. As malnourished children develop into adults, the disease burden established in childhood remains with them, leading to adult morbidity and decreased life expectancy. This decreases the adult’s capacity to contribute economically to the family, reinforcing poverty and completing the loop. Note that this loop contains two negative and three positive polarities and is a reinforcing loop because the negatives counteract each other. Adequate child nutrition is also diminished by depletion of natural resources, particularly in rural settings where foraging or hunting provides food sources.

Another important loop exhibiting reinforcing behaviour is R2 (purple/red), which shows the effect of family size on malnutrition [45]. The new loop connectors are shown in purple; poverty reduces access to modern family planning, increasing family size, which decreases the likelihood of adequate child nutrition; the loop is then completed by tracing the red arrows around the common linkage through malnutrition/stunting > child morbidity > adult morbidity > poverty.

Loop R3 (dark green/red) shows how malnutrition/stunting reduces children’s education through impaired cognitive ability and school absences [73] which, as children grow into adults, has a detrimental effect on levels of adult education. Increasing adult education supports adequate child nutrition and vice versa. The reinforcing loop is completed by the link back to malnutrition/stunting shown by the red arrow. Loop R4 (green/dark green) directly connects adult educational attainment to children’s educational attainment. Better educated adults are more likely to value and prioritise the education of their children. If child education levels increase, a virtuous circle of education level improvement is created; if they decrease, the reverse happens. Loop R5 (starting with orange dotted connector) shows how cultural norms, which are generally challenged by increasing levels of education, reinforce both the expectation of and desire for larger families [7]. The loop continues with a connection to adequate child nutrition (purple) and can be traced through malnutrition/stunting to child education and adult education. In some LDCs, a cultural norm is a lack of food priority for children [77]; this too is challenged by education. Potential opportunities to reverse negative reinforcing loops are child nutrition and education interventions.

#### 3.5.2. WASH-Related Disease Loops

**Figure 3:**
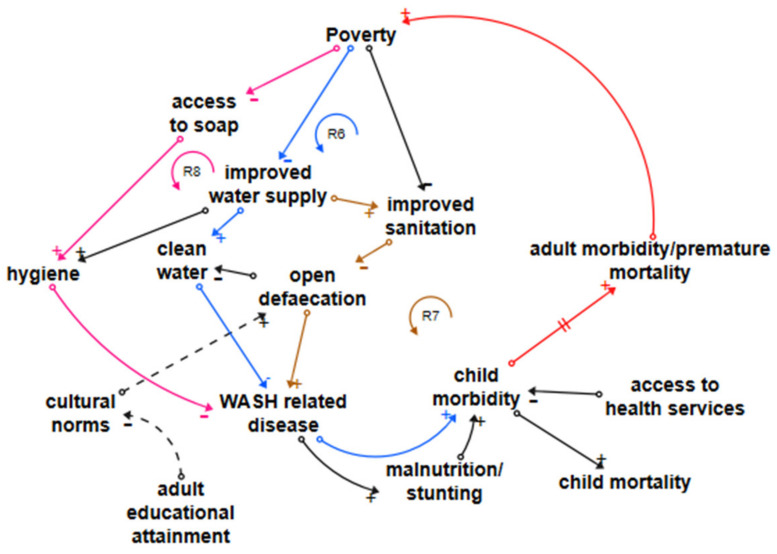
Loops influencing WASH-related disease.

Enteric disease, including diarrhoeal disease and typhoid, is estimated to be 95% attributable to inadequate WASH in LDCs [31,89]. Skin diseases, responsible for almost no child mortality but an estimated 9.9% of lifetime morbidity [31], are influenced by inadequate WASH and overcrowding. Loop R6 (blue/red) in Figure 3 shows the reinforcing loop connecting water access, clean water, and WASH-related disease, which includes enteric disease and skin diseases [3,18,34,35,36,37,38,39,40,41,42]. If an LDC’s government or external organisations do not provide a service, people and communities living in poverty, whether in informal settlements or a rural setting, do not have the resources to improve their own water supplies. Sufficient accessible clean water reduces all WASH-related disease. Improved water access is also a prerequisite for most improved sanitation services [60,61,62]. Loop R7 (brown joining red) shows the dependency of improved sanitation on improved water supplies. Improved sanitation is a prerequisite for the reduction or elimination of open defaecation, but poverty reduces the ability of communities to maintain sanitation facilities, and in some LDCs, there are powerful cultural traditions that impede a transition away from open defaecation. Education is required to support understanding of the benefits, as illustrated by the dotted lines linking adult education with cultural norms and open defaecation. Exposed faeces support the spread of pathogens, which increase WASH-related disease.

Loop R8 (pink joining red) shows how poverty reduces access to soap through a lack of resources to purchase it [18]. Adequate hygiene, including hand hygiene after defaecation and before food preparation, requires soap but also improved water access as in most cases, a natural clean water source is not close enough to the household to provide adequate water for hygiene purposes. In common with the loops shown in Figure 2, enteric disease increases child morbidity as repeated acute illness leads to chronic disease and later, either directly or through increasing malnutrition and stunting, to adult morbidity. Leverage points to reverse negative reinforcing loops are water and sanitation interventions combined with education, both general and specific.

#### 3.5.3. Air Pollution-Related Disease Loops

**Figure 4:**
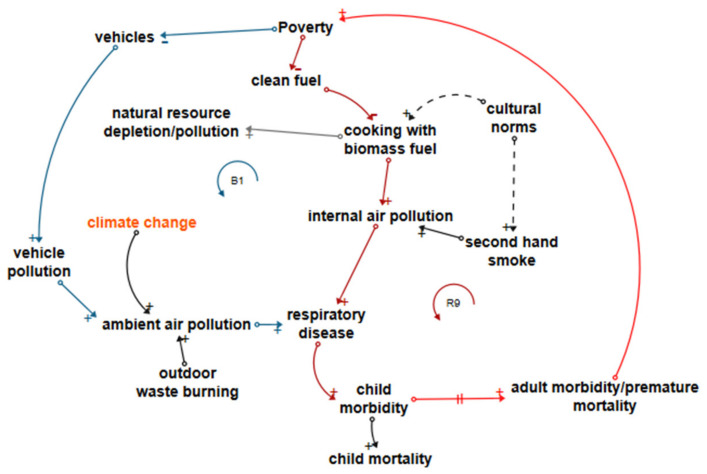
Loops influencing air pollution-related disease.

Figure 4 shows loop R9 (dark red joining red), a reinforcing loop linking the use of biomass fuel and respiratory disease, responsible for 14.7% of childhood mortality and 6.7% of morbidity in LDCs It illustrates how poverty reduces a household’s ability to acquire clean fuel and equipment with which to use it [7], forcing households to create internal air pollution through the use of biomass fuel, which is often available with no financial outlay to rural families (although it depletes natural resources, which in itself has consequences for the health of the environment and for the ability of the environment to provide for children’s nutrition). The respiratory disease burden of both mortality and morbidity with its roots in childhood is possibly one of the most difficult to address; long lead times mean that policymakers do not necessarily relate adult health consequences to lung damage sustained in childhood. A move away from biomass fuel requires not only clean fuel availability and affordability but also a willingness to embrace new ways of cooking, supported by an understanding of the health implications [87]. As with WASH-related disease loops, adult morbidity and premature mortality reinforce poverty.

Loop B1 (dark blue joining dark red then red) describes a balancing loop between poverty and vehicle pollution. If poverty increases, the number of vehicles reduces. The converse of this is that reducing poverty increases vehicle pollution and increases child and adult respiratory disease [88]. In this case, the positive health effects of poverty reduction are offset by a negative health effect of unsustainable development. The assumption here is that vehicles produce pollution; in LDC, vehicles are likely to be old and heavily polluting and are often exported from higher-income countries whose stricter regulations they no longer meet. Leverage points are air pollution reduction and clean cooking interventions combined with education.

#### 3.5.4. Vector-Related Disease and Skin Disease Loops

**Figure 5:**
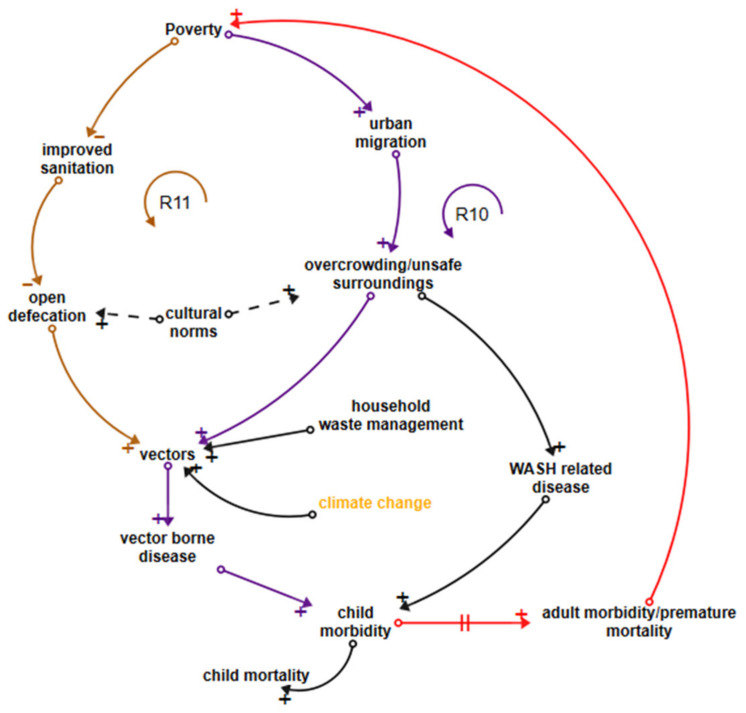
Loops influencing vector-related disease.

Vector-related disease accounts for an estimated 10% of child mortality and 9% of lifetime morbidity in LDCs [31]. Dengue fever is a growing urban problem whilst malaria still claims the greatest number of children’s lives in both rural and urban settings, particularly in African LDCs [18,51]. Loop R10 in Figure 5 (dark purple joining red) shows links between poverty, urban migration, overcrowding, numbers of vectors due to inadequate household waste management services, and vector-related disease. Overcrowding, particularly in informal settlements, is influenced in many LDCs by cultural obligations to house extended family and also contributes to skin disease transmission, WASH-related disease, and the spread of infectious diseases [54]. Loop R11 (brown joining dark purple and red) shows how poverty negatively influences improved sanitation, linking to open defaecation and increased numbers of vectors. Leverage points are in improving the built environment, waste management services and reduction of vector habitat as well as the WASH-related leverage points discussed earlier.

#### 3.5.5. Neonatal Disease Loops

**Figure 6:**
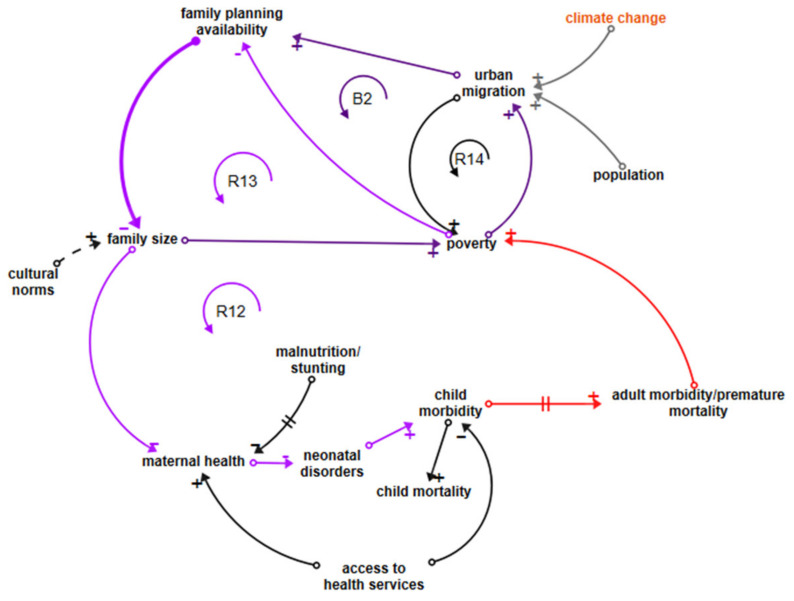
Loops influencing neonatal disease.

Loop R12 in Figure 6 is depicted in purple, linking poverty to family planning availability, family size, maternal health, neonatal disorders, and child morbidity. The loop continues in red through to adult morbidity and poverty. Neonatal disorders cause 28.7% of child mortality [31], with an estimated 20% attributed to the environment [89], but this percentage does not include the influence of maternal health and nutrition and access to health services, so one could argue that the total environmental attribution should be higher. CEH discussions do not generally include the influence of family size and maternal health on neonatal disorders or children’s health in general [45]; we believe that the CLD makes a case for doing so. A shorter reinforcing loop R13 also associates poverty with lower access to family planning availability, thus supporting increased family sizes.

Balancing loop B2 (dark purple/purple) depicts poverty driving migration from rural to urban or peri-urban areas. Access to modern family planning services is improved by moving to an urban area, with large variations between LDCs. This access acts to reduce family sizes, which helps to lift families out of poverty. However, urban migration in LDCs is increasingly forced by climate change and population growth, which leaves families unable to survive in rural areas, and another small reinforcing loop, R14, linking urban migration to increasing poverty, shows poverty and child malnutrition increasing in urban/peri-urban areas [83], counteracting the balancing effects of smaller families.

#### 3.5.6. Population Growth Loops

**Figure 7:**
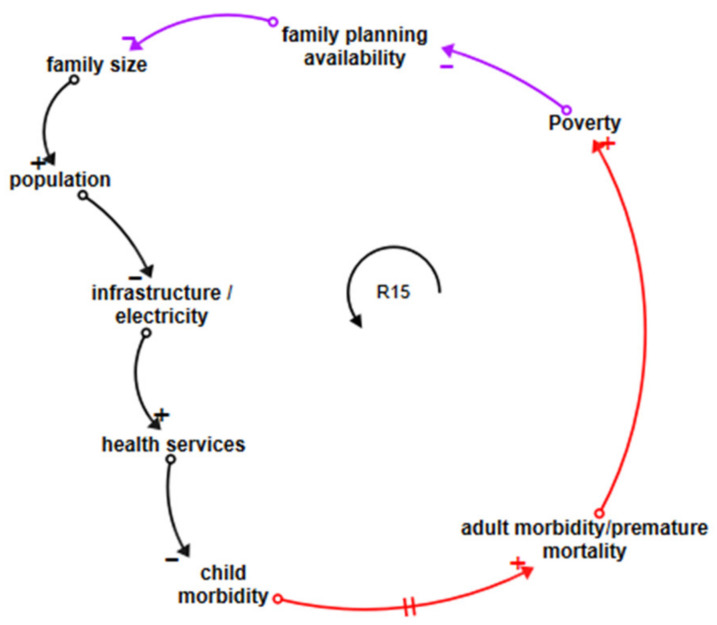
Loop influencing population growth.

Figure 7 shows that loop R15, starting in purple and moving through black and red, links household poverty with lower family planning availability and population growth. A larger population puts more demands on infrastructure, reducing the resources available per capita, whether for roads, improved water supplies, or health services. Improved health services reduce child morbidity and vice versa. Child morbidity leads to adult morbidity, which reinforces poverty, completing the loop. This vicious circle, which connects the situation of individual families to broad population growth and its socioeconomic impacts, and the situation in LDCs to the broader economic issues, is not, as far as we are aware, discussed in the CEH context even though lower per capita resources clearly have the potential to negatively affect children’s health outcomes.

### 3.6. Leverage Points

A key environmental determinant of health is sufficient and available clean water supplied close to the household to deliver not only safe potable water but also water for hygiene and sanitation purposes. This is well known, but Figure 2 reinforces the need for this leverage point to be supported by education, both general and specific, to support the uptake of water and sanitation interventions, to support cultural change where needed to overcome traditions and taboos that work against uptake, and to support the maintenance of WASH infrastructure. Reductions in both WASH-related and vector-borne diseases are the potential results. An estimated 60% of WASH benefits come from the elimination of open defaecation in communities [60], but this can be hard to achieve because of the many prerequisites.

Air pollution, both internal air pollution (IAP) and ambient air pollution (AAP), is an environmental determinant of health in LDCs, and interventions that support the uptake of clean cooking are leverage points for children’s lifetime health but need to be supported by education and community engagement. The relative cost of fuel is important; in rural areas, as long as biomass fuel is free, interventions that include education and a supply of clean cookstoves are unlikely to deliver improvements to households living in poverty without ongoing financial support. Leverage points for the reduction of AAP, particularly in urban areas, are vehicle emission reduction and regulation and household waste management interventions to reduce burning.

The growth of urban and peri-urban informal settlements, reinforced by poverty-related and climate change-driven migration, threatens children’s health through overcrowding and lack of infrastructure. Interventions that improve services, including waste management to informal settlements, are leverage points for children’s health.

All the major causal loops that include child morbidity connect through to adult morbidity and reinforcement of poverty. It follows that poverty reduction in LDCs will improve health outcomes unless it is countered by negative health effects such as increases in air pollution. However, continuing population growth, which effectively reduces infrastructure and health services available at an individual level and puts pressure on natural resources, reinforces poverty. Leverage points to address population growth are interventions in education and family planning. One successful intervention in LDCs has been immunisation programmes; these have delivered significant reductions in child mortality globally but have only increased population growth in LDCs.

Family planning availability has not been considered in the CEH field as one of the tools for improving children’s health in LDCs, but the impacts on maternal health, neonatal outcomes, children’s nutritional health, and access to health services are insight from this CLD and support a case for its inclusion as a leverage point.

## 4. Discussion

### 4.1. Application of the CLD

The CLD demonstrated in this paper is a system with many reinforcing causal loops that explain current behaviours of the CEH system through the interaction of a selected suite of endogenous variables [28]. Building a CLD based primarily on literature-derived variables is unusual; a more common approach is to collaboratively build or modify a CLD from participatory discussions with stakeholders [90]. This can either be done in community settings, with groups of policymakers/influencers or both, and can be a powerful tool for effective engagement in LDCs, lowering the risk of policy failure due to lack of cultural understanding [91].

Nevertheless, our work creates a better understanding of the often unsighted influencing loops that connect the environmental, social, and economic domains, highlighting and reinforcing known leverage points such as the need for education, cultural awareness, and community engagement if interventions are to yield positive results. It also shows how compounding delays reduce awareness of health outcomes; for example, the time delay between children’s respiratory clinic visits and adult respiratory-related mortality does not result in air quality policy interventions in LDCs because more immediate concerns dictate policy priorities.

These insights, particularly the links between population growth and children’s health, give us a big picture understanding of the issues facing LDCs and emphasise the need for support from more developed countries. There are many agencies active in LDCs delivering individual aid-based interventions, but the CLD highlights the need for collaboration across sectors to avoid suboptimal outcomes or unintended consequences. It also shows how LDCs, with external support, need to address poverty as a structural determinant of health, possibly in the context of the SDGs.

The selection of causal loops for further discussion in Section 3 does not mean that other disease groups, e.g., injuries, are unimportant and should not be analysed. One limitation of a CLD is that it is qualitative only; the choice of loops to discuss was based on quantitative information from Table 1. Similarly, disease groups such as mental health without sufficient literature-based evidence for links to the environmental, social, and economic domains in LDCs should not be ignored and point to a research gap.

The discussion of leverage points in Section 3.5 shows some examples of how interventions could change health outcomes. From a CLD perspective, interventions can change the polarity of reinforcing loops from detrimental to beneficial outcomes. This CLD is necessarily high-level; using the tool to focus on a specific EH problem, environment, or socioeconomic setting enables greater depth and more specific insights, which can guide decision-making about policy setting and practice.

### 4.2. Potential Application for Systems Science in CEH

The complex problem of poor CEH in LDCs cannot be solved solely through linear cause and effect approaches to problem-solving, such as the provision of water infrastructure to reduce WASH-related disease. A systems science approach has real potential to support decision-making in research as well as policy setting and practice [26]. In a quantitative systems science approach, dynamic simulation models are developed from a CLD and explicitly quantify relationships between influencing variables and describe their rates of change over time. Such models can then be used to simulate and compare the impacts of different policies and practices over time and to identify potential feedbacks, limitations, and inhibitors. Specifically, interventions can be simulated, and the levels of investment required to change the polarity of reinforcing loops and the time delays can be estimated in combination with a range of different assumptions.

Using these methods, we could, for example, estimate how child mortality and morbidity over the last 30 years will change into the future considering multiple and heterogenous influence variables and not, for instance, just the size of the investment or the potential uptake by the community. Similarly, we could extend the modelling to link adverse child health outcomes in LDCs to subsequent adult health outcomes [92], gaining a greater understanding of how morbidity with its origins in childhood is influencing adult health expectations. Many more influences can be defined and modelled. For example, the extent to which reduced child mortality contributes to population growth, which then reinforces poverty. Aid-funded interventions in infrastructure, for example, health service facilities or improved water supplies, will have a positive impact on children’s health outcomes, but we can now model how population growth and urban densification stretch these resources to the limit when considering the influencers from all domains.

To be useful to decision-makers and give meaningful results, a model would need to be built for a specific country or area and stratified into urban and rural segments. Lack of data in LDCs may make this task more difficult, but systems science can overcome such limitations to a degree by incorporating expert opinion and data from similar domains (e.g., other LDCs and proxies) into models.

## 5. Conclusions

Understanding is merely a starting point; delivering scientific recommendations that lead to action and sustained progress is surely the most important goal of CEH research. Systems science, currently underutilised in this field, can make an important contribution in all CEH settings, including LDCs. A CLD is a powerful tool in its own right for exploring and recognising the many interconnections between the environmental, social, and economic domains and their influence on children’s health outcomes and creating a shared understanding, and is also a first step in a quantitative dynamic modelling process. Our CLD shows the need to include a policy on population growth, family size, and family planning availability as an influencer of children’s health.

CEH most often focuses on environmental influences on health, and our CLD approach demonstrates how these should be augmented with social and economic influences and shows the impacts of poverty, low levels of education, and inadequate infrastructure on children’s health in LDCs.

## Figures and Tables

**Table 1:** Child mortality and morbidity for all least developed countries (LDCs) [31].

Child Mortality Disease Group	U15 ^1^ Deaths Rank	U15 Deaths %	U5 ^2^ Deaths Rank	U5 Deaths %	Child Morbidity Disease Group	U15 YLD ^3^ Rank	U15 YLD %	U5 YLD Rank	U5 YLD %
Neonatal disorders	1	28.7	1	31.6	Nutritional	1	26.6	1	35.0
Enteric disease	2	15.1	3	14.5	Skin/subcutaneous diseases	2	9.9	4	8.4
Respiratory disease	3	14.7	2	15.2	*Other NCDs*	3	9.8	2	12.0
NTDs ^4^ and malaria	4	10.2	4	10.1	*Mental disorders*	4	9.6	8	3.2
Other infectious diseases	5	9.0	5	8.7	NTDs and malaria	5	9.0	5	8.1
*Other NCDs*	6	8.3	6	8.6	Respiratory disease	6	6.7	7	6.5
*HIV/AIDS*	7	3.0	7	2.7	Neonatal disorders	7	6.3	3	9.3
Unintentional injuries/transport injuries	8	4.2	9	2.9	Neurological disorders	8	5.8	9	2.9
Nutritional	9	2.4	8	2.5	Enteric disease	9	4.3	6	6.7
*Neoplasms*	10	1.1	10	0.7	*Sense organ diseases*	10	3.0	-	-
					Other infectious diseases	-	-	10	2.8

^1^ children aged under 15 ^2^ children aged under 5 ^3^ Years Lived with Disability ^4^ Neglected Tropical Diseases. Causes of mortality/morbidity from Level 2 ICD codes [31]: Morbidity is measured in years lived with disability. Neonatal includes maternal/neonatal disorders. Other infectious diseases include meningitis, measles. Other NCDs include congenital birth defects and sudden infant death syndrome. Enteric diseases include diarrhoea and typhoid. NTDs include dengue fever, yaws, trachoma, helminths including hookworm, ascariasis, and trichuriasis. Skin diseases include scabies and fungal skin diseases. Respiratory includes upper and lower respiratory infections, tuberculosis, and chronic respiratory disease. Mental disorders include intellectual disability. *Italics: denote disease groups excluded from further analysis.*

**Table 2:** Children’s health outcome variables with influencing links.

Variable	Influenced by	+/−	Influences	+/−	Source
**Water, sanitation and hygiene (WASH)-related disease**—includes enteric diseases of which diarrhoea is the most prevalent, parasitic NTDs including hookworm and schistosomiasis, all of which have strong causal links with polluted water, open defaecation, and inadequate hygiene. Helminth infections also increase the risk of diarrhoeal disease. Skin diseases, e.g., scabies, yaws are strongly associated with inadequate hygiene measures and overcrowding.	Clean water Open defaecation Hygiene Overcrowding	− + − +	Malnutrition/stunting Child morbidity/mortality	+ +	[3,18,34,35,36,37,38,39,40,41,42]
**Malnutrition/stunting**—is the biggest contributor to YLD in LDCs. The effects of a lack of nutritious food are compounded by acute and chronic diarrhoeal disease and helminth infections. A lack of micronutrients in childhood can also predispose to obesity in later life, increasing the risk of NCDs.	Adequate child nutrition WASH-related disease	− +	Child morbidity/mortality Child education	+ − +	[3,18,43,44,45]
**Respiratory disease**—is caused by both internal air pollution (IAP) and ambient air pollution (AAP) exposure in LDCs. Rural exposure is primarily through IAP whilst urban/peri-urban exposure is to both IAP ^1^ and AAP ^2^. Overcrowding increases transmission of infectious respiratory diseases such as TB.	Internal air pollution (IAP) Ambient air pollution (AAP) Overcrowding	+ + +	Child morbidity/mortality	+	[3,18,34,46,47,48,49]
**Vector-borne disease**—such as malaria, which is the fourth largest contributor to child mortality. Dengue fever is a growing threat in urban areas. Other vector-borne diseases include chikungunya fever, Zika virus, Chagas disease, and leishmaniasis. Infection cycles are perpetuated by mosquitos as well as flies, which use faeces in their breeding cycles.	Vectors	+	Child morbidity/mortality	+	[3,4,18,50,51]
**Childhood injury including traffic injuries**—is a risk of unsafe surroundings. Whilst injury is perceived mainly as a problem of an unsafe built environment, remoteness from health resources worsens the prognosis after injury.	Overcrowding Unsafe surroundings	+ +	Child morbidity/mortality	+	[52,53,54]
**Child morbidity/mortality**—is increased by inadequate access to health services. In all LDCs, shortages of health personnel, infrastructure, pharmaceutical supplies, and medical equipment are a limitation; in rural and remote areas, these are exacerbated by the need to travel long distances with minimal transport infrastructure. Maternal poor health leads to suboptimal birth outcomes such as fetal growth restriction, low birthweight, and suboptimal breastfeeding.	Disease group mortality/morbidity Health services Maternal health	+ − −	Adult morbidity/premature mortality	+	[1,7]
**Maternal health**—Female childhood malnutrition, insufficient nutrition for pregnant women and mothers in some LDCs, large families, and inability to plan and space families all contribute to poor maternal health.	Malnutrition/stunting Family size	− −	Child morbidity/mortality	+	[45,55]
**Adult morbidity/premature mortality**—is a direct result of chronic adult illness with its roots in childhood, reducing lifespan (average 63 in LDCs versus global lifespan of 73) and lifetime economic contribution in LDCs.	Child morbidity	+	Poverty	+	[56,57,58]
**Neonatal disorders**—lead to neonatal deaths and, for survivors, increased mortality risk and morbidity such as stunting, with subsequent adult mortality and morbidity implications.	Maternal health IAP	− +	Child morbidity/premature mortality	+	[18,45,55]

^1^ Internal Air Pollution ^2^ Ambient Air Pollution.

## Data Availability

Publicly available datasets were analyzed in this study. The data can be found at http://www.healthdata.org/gbd/2019 (accessed on 25 January 2021).

## Supplementary Materials

The following are available online at https://www.mdpi.com/1660-4601/18/6/3010/s1, Supplementary S1: CLD creation and terminology.
