# Supplementary material for: Applications of Systems Science to Understand and Manage Multiple Influences within Children’s Environmental Health in Least Developed Countries: A Causal Loop Diagram Approach
Source: Int J Environ Res Public Health. 2021 Mar 15;18(6):3010. doi: 10.3390/ijerph18063010 (PMC8001252; doi:10.3390/ijerph18063010)
Supplement: Supplementary file 1 [file ijerph-18-03010-s001.pdf]

Supplementary Material S1

S1.1 Terminology used in the article

Systems science, also referred to as systems thinking, uses terminology which may be unfamiliar. The terms used in the article are further defined here:

- Mental model – a set of beliefs, values and assumptions which underly and explain why things work as they do. A mental model is an explanation of a person or a group's thought process about how something works in the real world. It is a representation of the surrounding world, the relationships between its various parts and a person's intuitive perception about his or her own acts and their consequences.
- *A Causal Loop Diagram is a representation of a 'mental model' and when it is developed or refined by a group it enables shared understandings and appreciation of others' perspectives to be gained and paves the way to agreement on what is significant or important;*
- Variable – an element, feature or factor that is liable to vary or change (Oxford English Dictionary). In the context of systems science, a variable can be almost any factor in a system; it may be quantitative e.g. population number or it may be qualitative e.g. cultural belief or happiness.
- Causal Influence / Cause-effect link / Influencing link – a relationship between two variables denoted by an arrow showing the direction of the influence. Normally a causal influence will be uni-directional e.g. cooking with biomass fuel causes air pollution.
- Domain – a specified sphere of activity or knowledge or a particular field of thought, activity, or interest.

S1.2 Causal Loop Diagram explanation

A Causal Loop Diagram consists of variables and influencing links which connect to form feedback loops. Whilst a CLD is a qualitative representation, the variables it uses can be both quantifiable (e.g. mortality, environmental measures) and qualitative (e.g. cultural influences, people's perspectives). These variables are linked by directional arrows which represent causal associations. Associations can be either reinforcing, denoted by **+** or opposing, denoted by **-** .

Figure S1 shows a reinforcing causal association between chicken and egg; more chickens lead to more eggs. The reinforcing association also means than less chickens lead to less eggs; i.e. an increase in the 'chicken' variable causes an increase in its linked variable and a decrease also causes a decrease in the linked variable.

Figure S1 also shows an opposing causal association between market competition and price; more competition leads to a price reduction and less competition leads to a price increase.

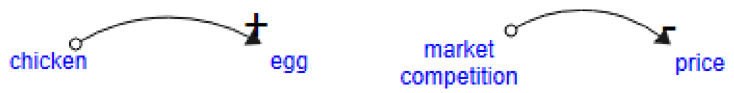

Figure S1: causal links

A causal loop is formed by the linking together of the variables in a closed connected path. Hash marks on a connector arrow denote a delay between cause and effect. Causal loops are either reinforcing or balancing. Reinforcing loops can be beneficial or detrimental; the 'vicious and virtuous' circles of everyday language. The feedback structure in a reinforcing loop generates exponential growth or decline.

Supplementary Material S1

Figure S2 shows a detrimental reinforcing loop associated with respiratory disease in LDCs. Most of the causal links are positive but there are two negative links; increased poverty is associated with a reduction of clean fuel usage and an increase in clean fuel usage is associated with less cooking with biomass fuel. A delay marker can be seen on the causal link between child morbidity and adult premature mortality; there is a lead time between illness with its roots in childhood and early death in adulthood.

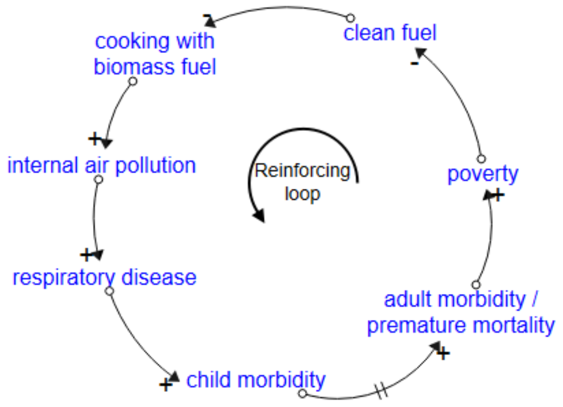

Figure S2: reinforcing loop

Balancing loops are self-regulating or counteracting. Figure S3 illustrates a balancing loop in an epidemic model which shows how, as the infected population grows, the dead or immune recovered population grows, lowering the susceptible population. As the susceptible population reduces, the infection rate drops.

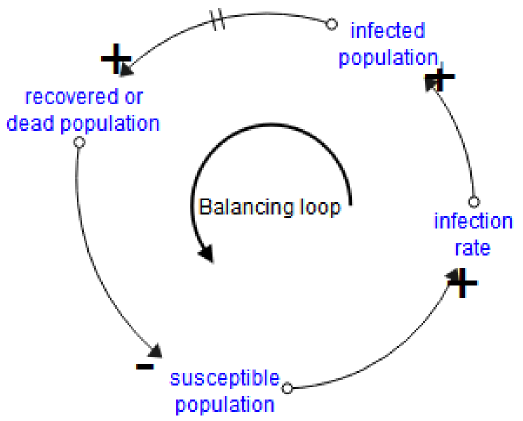

Figure S3: balancing loop

How can one work out whether a loop is reinforcing or balancing? Loops are made up of reinforcing (plus sign on arrow) and balancing associations (minus sign on arrow). Two balancing associations cancel each other out, therefore reinforcing loops contain an even number of minus signs and balancing loops an odd number of minus signs. All the variables shown in Figures S2 and S3 are endogenous; arising from within, which means that they are all influenced by other variables in the model.

Variables in a CLD can also be exogenous; arising from outside the boundary of the model. Exogenous variables are the assumed factors which are not explained by the interactions within the model. In practical terms, this means that as exogenous inputs cannot be influenced by other variables in the model, the focus for interventions in the context of the model should lie elsewhere.

Figure S4 shows remoteness as an exogenous variable that inputs into a CLD; it influences other variables in the CLD but is a geographic characteristic which cannot be changed. Remoteness in this examples is therefore at the start of the chain and has no feedback loops.

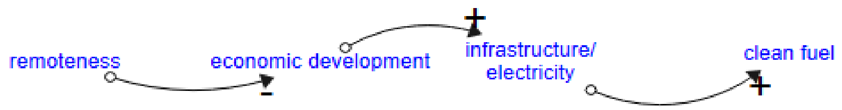

Figure S4: Exogenous input
